# Telemetry without collars: performance of fur- and ear-mounted satellite tags for evaluating the movement and behaviour of polar bears

**DOI:** 10.1186/s40317-024-00373-2

**Published:** 2024-07-15

**Authors:** Tyler R. Ross, Gregory W. Thiemann, B. J. Kirschhoffer, Jon Kirschhoffer, Geoff York, Andrew E. Derocher, Amy C. Johnson, Nicholas J. Lunn, David McGeachy, Vicki Trim, Joseph M. Northrup

**Affiliations:** 1 Department of Biology, York University, Toronto, ON Canada; 2 Faculty of Environmental and Urban Change, York University, Toronto, ON Canada; 3 Waterfall Innovation, Bozeman, MT USA; 4 Stillwater, MN USA; 5 Polar Bears International, Bozeman, MT USA; 6 Department of Biological Sciences, University of Alberta, Edmonton, AB Canada; 7 Ecofish Research Ltd., Courtenay, BC Canada; 8 Environment and Climate Change Canada, Edmonton, AB Canada; 9 Department of Agriculture and Resource Development, Manitoba Sustainable Development, Thompson, MB Canada; 10 Ontario Ministry of Natural Resources and Forestry, Peterborough, ON Canada

**Keywords:** Telemetry, Fur tag, Hair tag, Polar bear, *Ursus maritimus*

## Abstract

The study of animal movement provides insights into underlying ecological processes and informs analyses of behaviour and resource use, which have implications for species management and conservation. The tools used to study animal movement have evolved over the past decades, allowing for data collection from a variety of species, including those living in remote environments. Satellite-linked radio and GPS collars have been used to study polar bear (*Ursus maritimus*) ecology and movements throughout the circumpolar Arctic for over 50 years. However, due to morphology and growth constraints, only adult female polar bears can be reliably collared. Collars have proven to be safe, but there has been opposition to their use, resulting in a deficiency in data across much of the species’ range. To bolster knowledge of movement characteristics and behaviours for polar bears other than adult females, while also providing an alternative to collars, we tested the use of fur- and ear-mounted telemetry tags that can be affixed to polar bears of any sex and age. We tested three fur tag designs (SeaTrkr, tribrush and pentagon tags), which we affixed to 15 adult and 1 subadult male polar bears along the coast of Hudson Bay during August–September 2021–2022. Fur tags were compared with ear tags deployed on 42 subadult and adult male polar bears captured on the coast or the sea ice between 2016 and 2022. We used data from the tags to quantify the amount of time subadult and adult males spent resting versus traveling while on land. Our results show the three fur tag designs remained functional for shorter mean durations (SeaTrkr = 58 days; tribrush = 47 days; pentagon = 22 days) than ear tags (121 days), but positional error estimates were comparable among the Argos-equipped tags. The GPS/Iridium-equipped SeaTrkr fur tags provided higher resolution and more frequent location data. Combined, the tags provided sufficient data to model different behavioural states. Furthermore, as hypothesized, subadult and adult male polar bears spent the majority of their time resting while on land, increasing time spent traveling as temperatures cooled. Fur tags show promise as a short-term means of collecting movement data from free-ranging polar bears.

**Supplementary Information:**

The online version contains supplementary material available at 10.1186/s40317-024-00373-2.

## Introduction

The movement of organisms is an essential component of life, shaped by ecological and biological processes often acting across multiple spatial and temporal scales [1–4]. While humans have tracked the movements of animals for millennia, in recent decades, the study of where and how animals travel through their environment has broadened our understanding of the factors influencing habitat selection and species distributions [5–8]. Analyses of animal movements provide opportunities to better understand these species–habitat relationships and improve predictions of space use and, ultimately, population dynamics, which depend on the spatial distribution of individuals.

Methods used to record animal movements have evolved over the past 50 years. Progressively smaller satellite-linked transmitters, along with advances in battery technology, have allowed for remote collection of data from an increasing variety of organisms, including cryptic and migratory species, and those living in remote environments, where direct observation is impractical [9, 10]. Associated increases in the spatial and temporal resolution of location data also afford refined insights into biological and environmental factors affecting animal movement. These advances have provided benefits for species conservation and management through the identification of critical habitat and elucidation of shifting movement patterns, such as changes in migration phenology and distribution in response to climate change [11–14].

Climate change poses a particular risk to some high latitude species due to the effects of Arctic amplification, whereby the Arctic is warming at a rate approximately four times faster than the global average [15–18]. Polar bears (*Ursus maritimus*) are marine carnivores that travel thousands of kilometers over large seasonal home ranges throughout the circumpolar Arctic [19–21]. Their movements are linked to sea-ice dynamics, as bears select for areas of high sea-ice concentration over the continental shelf where ocean productivity and prey availability are high [22, 23]. Due to the dynamic nature of Arctic sea ice, polar bears change their movements in response to sea-ice drift and seasonal fragmentation [24–27]. Over the past several decades, climate-related reductions in sea ice have altered polar bear movements, resulting in shifts in home range sizes and distribution [28–30], increased use of terrestrial habitats [31–33], and observations of long-distance swimming [34–36]. In the southern portion of polar bear range, sea ice undergoes an annual freeze–thaw cycle and bears are forced ashore during the ice-free season [37–39]. During this time, some bears remain close to the coast, while others move inland to maternal denning habitat or refugia to avoid disturbance from other bears [40–43]. In Hudson Bay, the duration of the ice-free season has continued to increase since the late 1970s, forcing bears to remain ashore and fast for progressively longer periods [23, 44–46]. Thus, understanding the movements and behaviours of polar bears in light of changes to sea ice is a critical conservation topic.

Since the late 1970s, satellite-linked radio and global positioning system (GPS) collars fitted around the necks of adult female bears have been the primary means of studying polar bear movements, distribution, behaviour, and habitat selection [47, 48]. In general, subadult bears are not collared due to the potential for growth-related injury, whereas adult males are rarely collared because the circumference of their neck exceeds that of their head, making collars likely to slip off [47, 49, 50]. Although studies of polar bear behaviour and habitat selection have used movement data from collared subadult bears (see [51] and [52]), both sample size and collar functional duration were limited in comparison to the studies examining adult female movements. Other attachment methods, including harnesses [53, 54], ear tags [55, 56], subcutaneous implants [50, 57], and adhesives [55, 58], have been used to temporarily affix transmitters to subadult or adult male polar bears with varying success [47]. For instance, ear-mounted transmitters deployed between 2007 and 2013 were functional for short durations, averaging approximately 70 days, whereas collars often transmit for several years [47, 56, 59]. Wiig et al. [56] noted several instances of injury from infection and forced removal of ear-mounted transmitters. The authors speculated that bears may have torn the transmitters from their ear, or the transmitters may have become caught on objects in their surroundings, resulting in observations of split ears. Based on these observations, the authors recommended the use of smaller and lighter ear-mounted transmitters, and adopting a non-permanent attachment system to prevent tissue damage. Thus, there is a need to refine attachment methods to ensure animal welfare and provide location data for subadult and adult male bears.

Although collars are the most reliable means of collecting multi-year polar bear movement data, in addition to the limits on which sex and age classes can be monitored with this method, there has been public concern, particularly from Indigenous communities, about possible negative physical and behavioural impacts. Specifically, concerns of possible injuries caused by collars becoming too tight, and impairment of a bear’s ability to successfully hunt seals have been raised [60–62]. Studies have demonstrated collars have no appreciable impact on polar bear movement rates, body condition, recruitment, or survival [55, 63], and their use has been critical for polar bear management [47]. However, there is an ongoing desire to refine polar bear research techniques to ensure that they are effective, minimally invasive, and respect the views of all stakeholders [60].

The limited amount of movement data collected from subadult and adult male polar bears suggest that there may be sex and age-class related differences in movement rates, behaviour, dispersal, and habitat selection [50, 52, 56, 64]; however, data are needed to better assess these differences. Thus, to increase knowledge of movements and associated behaviours of polar bears other than adult females, while providing an alternative to collars, we tested the use of fur- and ear-mounted telemetry tags that can be affixed to polar bears of all sex and age-classes. Specifically, we tested three different designs of fur tags and compared their performance, in terms of both retention time and error resolution, to ear tags that were smaller than those used by Wiig et al. [56]. Furthermore, using both fur and ear tags, we collected location data from subadult and adult male polar bears to examine their movement patterns and associated behavioural states during the ice-free season along the Hudson Bay coast in Canada. We hypothesized that subadult and adult male polar bears would spend the majority of the ice-free season resting, increasing their time spent traveling as temperatures decreased and sea ice in Hudson Bay began to reform in early winter.

## Materials and methods

### Study area

**Fig. 1:**
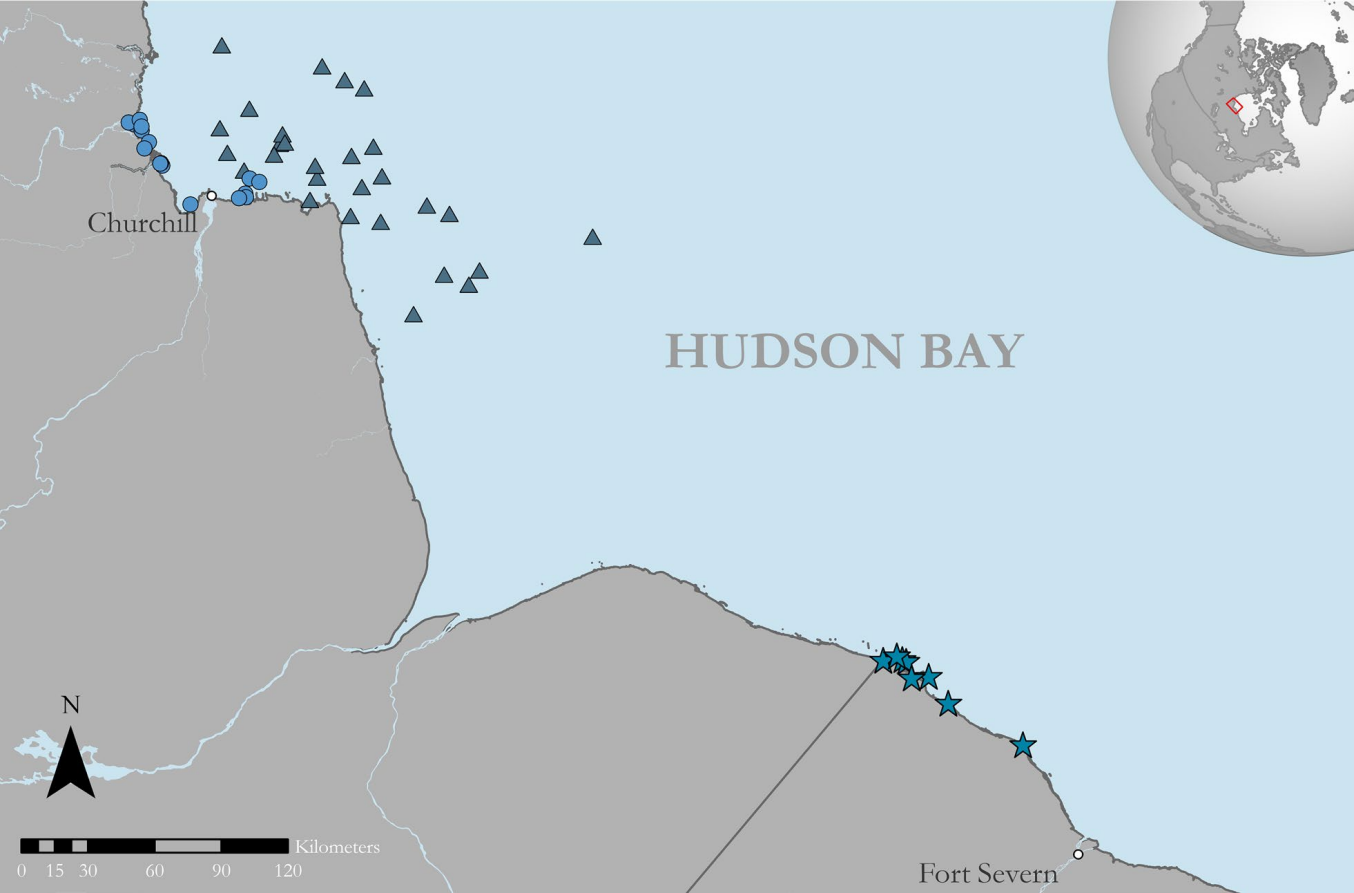
Portion of the Hudson Bay coastline encompassing the area where subadult and adult male polar bears were equipped with fur- and ear-mounted tags between 2016 and 2022. Triangles denote locations of spring ear tag deployments (*n* = 28; 2017–2019, 2022); circles denote fall ear tag deployments (*n* = 16; 2016–2021); and stars denote fall fur tag deployments (*n* = 16; 2021–2022). Several fur tags were affixed to bears at the same locations resulting in overlapping points

This study occurred along the southwestern coast of Hudson Bay, Canada, bounded roughly by Fort Severn, Ontario in the southeast and near Churchill, Manitoba in the northwest (Fig. 1).

Most of the terrestrial area falls within the Hudson Bay Lowlands ecoregion, which includes extensive marine beaches and coastal mudflats that become exposed during low tide [65, 66]. Further inland, beach sediments and coastal vegetation transition to areas dominated by mosses and lichen interspersed with large patches of willow (*Salix* spp*.*), alder (*Alnus* spp.), and dwarf birch (*Betula glandulosa*)*.* In the southern portion of the study area (i.e., below treeline), tundra and wetland vegetation transition to black spruce (*Picea mariana*) and white spruce (*P. glauca*) boreal forest [65–67]. Hudson Bay remains frozen for most of the year, but becomes ice-free between August and November. Sea ice typically begins reforming along the western coast in December and reaches its maximum extent and thickness in March or April [68, 69].

### Tag design and application

**Fig. 2:**
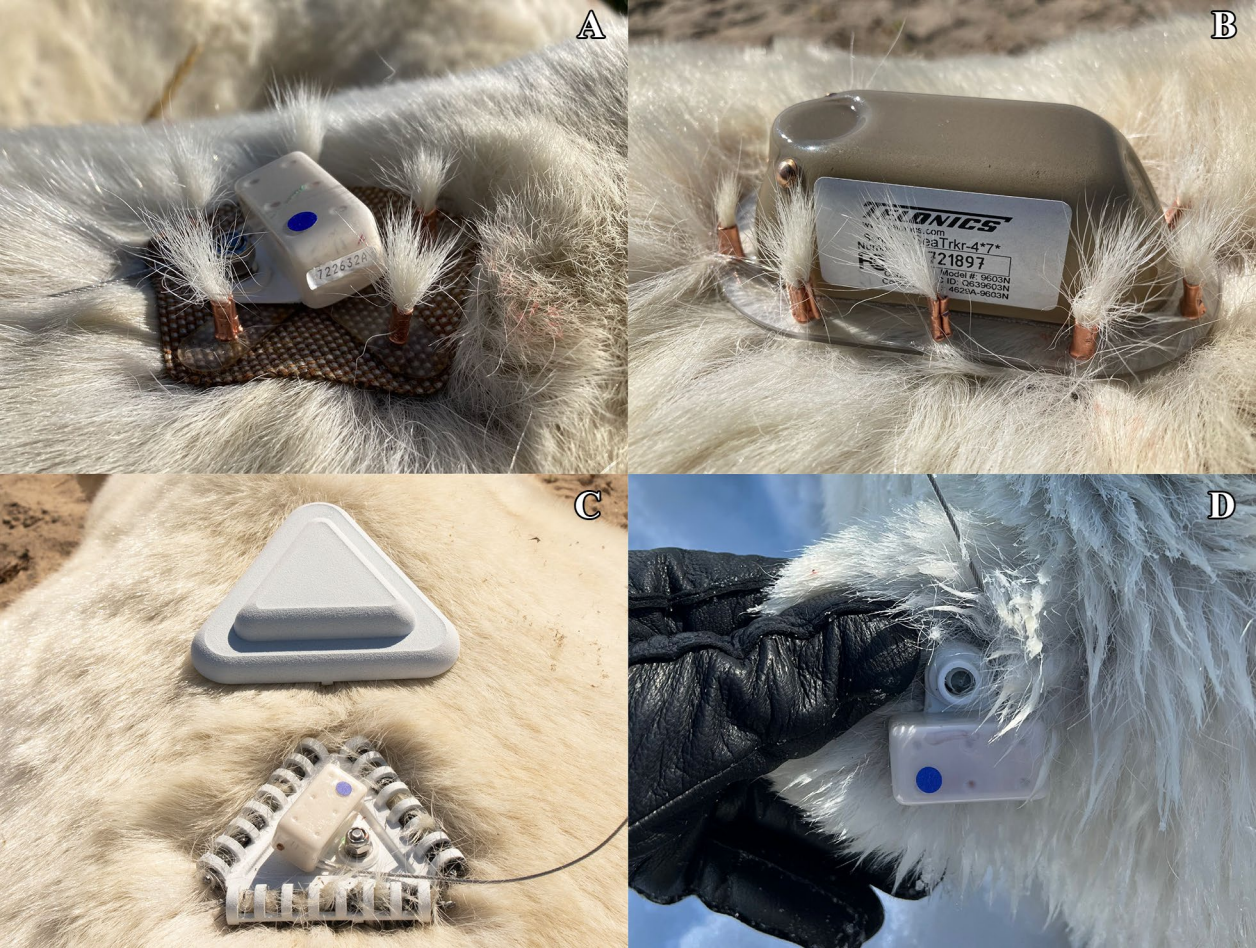
Ear tag and three different designs of fur tags attached to free-ranging subadult and adult male polar bears on the sea ice or along the coast of Hudson Bay between 2016 and 2022. Pentagon (**A**) and SeaTrkr (**B**) tags were mounted using copper ferules crimped around several clumps of hair, whereas tribrush tags (**C**) were affixed by ensnaring guard hairs in three nylon brushes secured in perforated tubes. Also shown is a radome cover that was placed atop each tribrush tag. Ear tags (**D**) were secured through a hole made in the ear using a 6-mm punch. For scale, the Argos transmitters (white rectangular cuboid with blue dot) pictured in **A**, **C**, and **D** measure 3.9 × 2.0 × 1.9 cm. The SeaTrkr tag pictured in **B** measures 10.3 × 4.5 × 3.6 cm

We tested three different fur tag designs. The first was made of a ballistic mesh (*n* = 3; 2021) or rubber (*n* = *3;* 2022) and strips of semi-rigid plastic backing cut into a pentagonal shape with holes punched into each of the five corners (Fig. 2A).

Using a cable puller, polar bear hairs were ensnared and pulled through each hole. A copper ferrule was then placed around the hairs and slid to the base of the tag, where it was crimped twice in orthogonal directions using pliers. Herein referred to as a ‘pentagon tag’, this tag measured 8.6 cm at the widest point and was equipped with a 3.9 × 2.0 × 1.9 cm, 26 g Argos Eartag Transmitter (ETA-2620; Telonics, AZ, USA), secured to an accessory bolt on the tag using a nut and locking washer. Combined with the ear tag transmitter and attachment hardware, each pentagon tag weighed a total of 51 g. The second tag design, commercially available as the SeaTrkr GPS/Iridium tag (Telonics, AZ, USA), was similar in design; however, it included an oval-shaped rigid plastic baseplate with ten equally spaced holes around its outside edge (Fig. 2B). It was attached to hairs in the same manner as the pentagon tag. The SeaTrkr tag was equipped with an Iridium-linked Telonics GPS SeaTrkr-4370 transmitter, which measured 10.3 × 4.5 × 3.6 cm and weighed 190 g. The 10 copper ferules used to secure the SeaTrkr tags weighed 8 g, for a combined total weight of 198 g. The third tag design, herein referred to as a ‘tribrush tag’, was a rigid plastic triangle measuring 10.3 cm at its widest point with perforated tubes spanning the length of each edge (Fig. 2C). After placing the tag firmly against a bear’s fur, a 12 mm × 75 mm nylon pipe brush was then inserted into each of the three tubes and twisted clockwise, ensnaring the bear’s hair in the brushes’ plastic bristles. The brushes were twisted until they collected enough hair that they could no longer be turned by hand, and the metal handles were cut flush with the base of the bristles. The same ETA-2620 transmitters used on the pentagon tags were attached to the tribrush tags, but were subsequently covered with a protective radome cover that clipped securely to the aforementioned perforated tubes. Combined with the transmitter, brushes, and radome cover, each tribrush tag weighed a total of 92 g.

The ear tags (ETA-2620; Telonics, AZ, USA) consisted of an Argos transmitter (ST-26; Telonics, AZ, USA) housed in a plastic casing with an integrated washer. Using specialized pliers, the tag and a separate friction-fit pin (Allflex, Rahway, NJ, USA) placed on the ventral side of the bear’s ear were clamped together, secured through a hole made in the ear using a 6-mm punch.

### Polar bear capture and tag deployment

During August and September 2021–2022, subadult (3–4 yr) and adult (≥ 5 yr) male polar bears were opportunistically located from a helicopter along the Hudson Bay coast near the Ontario-Manitoba border (Fig. 1). Bears were chemically immobilized via remote injection using zolazepam–tiletamine (Zoletil®; Virbac, France) following Stirling et al. [70]. Ages were determined using counts of cementum growth layers from a vestigial premolar extracted during handling or records from previous capture [71]. Each bear received one of the three fur tags, all of which were secured to hairs above the thoracic spine, immediately posterior the scapulae (Fig. 2). For two of the tribrush tags, a fast-cure two-part epoxy (3 M Scotch-Weld Epoxy Adhesive DP100) was applied to the ensnared hairs to assess the influence of supplementary adhesive on tag retention time. Ear tags were deployed on polar bears immobilized on the Hudson Bay sea ice during spring 2017–2019 and 2022 using the same capture and handling protocols.

Additional ear tags were deployed between September and November 2016–2021 on subadult and adult polar bears captured within or near Churchill, Manitoba as part of ongoing operations of the Polar Bear Alert Program [72, 73]. Polar bears deemed a threat to human life or property were captured by Manitoba government staff via remote injection using zolazepam–tiletamine. Sex and reproductive status were determined during handling. Age was verified using the same procedures noted above and included examination of tooth eruption patterns for dependent offspring [71]. Argos-linked tags, including pentagon and tribrush fur tags were programmed to record locations every 4 h, whereas GPS/Iridium SeaTrkr tags were programmed to record locations every 2 h. Based on these settings, and assuming a mean ambient temperature of 10 °C, batteries for the Argos- and GPS/Iridium-linked tags were expected to last approximately 5–6 months and 11 months, respectively.

Research protocols were reviewed and approved annually by the animal care committees of Environment and Climate Change Canada (Prairie and Northern Region), the Ontario Ministry of Natural Resources and Forestry, University of Alberta, and York University, and followed the guidelines of the American Society of Mammalogists for the use of wild mammals in research [74] and the Canadian Council on Animal Care (www.ccac.ca).

### Data preparation and statistical analyses

We evaluated two performance metrics for each tag design. First, we measured functional duration (i.e., the number of days transmitters/receivers remained active while attached to a bear). A tag was deemed inactive once it stopped providing location data due to malfunction or battery exhaustion, or when it provided consistent inactivity signals (> 3 consecutive fixes), indicating detachment. Tags were programmed to enter inactivity mode following a period of sustained lack of movement lasting ≥ 12 h, after which an automatic notification was sent indicating the tag had stopped moving. Second, we measured horizontal error estimates and error classes associated with each point location recorded using GPS- and Argos-linked transmitters, respectively. Horizontal error estimates for GPS/Iridium transmitters are expressed in meters and represent the radius of a circle surrounding each reported GPS position within which the transmitter was likely located. Each Argos location was assigned one of six possible error classes (3, 2, 1, 0, A, B; in order of decreasing accuracy) based on the number of messages transmitted to passing satellites. Locations derived from ≥ 4 messages have small horizontal error (< 250 m for Class 3; 500 m for Class 2; 1500 m for Class 1; and > 1500 m for Class 0), while those derived from < 4 messages cannot be estimated [9, 75].

After determining the tags’ functional duration and horizontal error using the full suite of data, we truncated the first 3 days of location data post-capture, as this is the approximate period during which polar bear activity and movement rates may be affected by immobilization [55, 63]. We then used an automated filtering routine in the argosfilter R package [76] to remove biologically implausible locations that implied movement rates ≥ 40 km h^−1^ between successive locations [77]. Polar bears have been observed running at speeds of 30–40 km h^−1^ for short durations, but sustain speeds of approximately 4 km h^−1^ over longer periods [78, 79]; therefore, this threshold represents a conservative upper limit intended to identify only extreme outlier observations [80]. Finally, locations were further subset to include only those observations recorded on land. Using hidden Markov models (HMM), we used these terrestrial locations to examine movements and associated behavioural states of subadult and adult male polar bears during the ice-free period in Hudson Bay. Forays into Hudson Bay (< 50 consecutive locations), whether during the open water season or as the sea ice was forming in early winter, were included as long as the bear returned to land during the same season before moving onto the sea ice for the winter. The remaining locations had sporadic temporal gaps longer than the programmed fix rates for both Argos- (4 h) and GPS/Iridium-linked tags (2 h). Considering HMMs require telemetry data to be provided at consistent intervals [81, 82], we standardized the data to consistent 4 h time intervals by interpolating missing Argos locations and rarifying GPS/Iridium locations using the R package crawl [83, 84]. There were also instances of longer gaps (> 12 h) in the data due to consecutive failed fix attempts. Interpolating telemetry data across longer gaps is problematic because it produces uncertainty in missing location estimates [85, 86]. Therefore, we segmented polar bear movement paths when temporal gaps between successive locations were > 24 h, thereby removing these intervals of missing data from analyses. Each track segment was treated as an independent time series arising from the same underlying statistical model, and because fitted parameters are common to all tracks, the same behavioural states influence movements both before and after the gaps, and any existing correlation was accounted for [86, 87]. Finally, individual segments with < 100 locations were omitted from analyses, because they provided little information about the transition between, and relative time spent in, different behavioural states [86].

We estimated the proportion of time subadult and adult male bears spent resting versus traveling using the R package momentuHMM, which uses discrete-time HMMs to infer latent behavioural states and associated transition probabilities from animal telemetry data [82]. Fundamentally, HMMs estimate distinct, unobserved behavioural states based on attendant movement characteristics, often using derived quantities such as step lengths (i.e., distance between successive point locations) and turn angles (i.e., directional change between subsequent steps) between consecutive telemetry locations [81, 88]. For instance, ‘foraging’ behaviour may be associated with movement paths characterized by short step lengths and large turn angles, indicating tortuous movements, whereas ‘transiting’ behaviour may be characterized by long step lengths and near-linear movement paths (i.e., near-zero turn angles). Here, we modelled step lengths and turn angles using gamma and von Mises distributions, respectively. Due to the sensitivity of HMMs to initial values when calculating maximum likelihood estimates for model parameters, we refit each model 50 times using different starting values for step length mean, step length standard deviation, turn angle mean, and turn angle concentration for the two behavioural states. Standardized Argos and GPS/Iridium data (i.e., regularized to consistent 4-h fixes) were pooled to increase sample size and inferential power. We also modelled state transition probabilities as a function of ambient temperature using hourly temperature data collected from the nearest permanent weather station, located in either Churchill, Manitoba or Fort Severn, Ontario (https://climate.weather.gc.ca/; accessed October 2022). State transition probabilities were modelled as a function of hourly ambient temperature using both a linear (*temp*) and quadratic term (*term*^*2*^), and were compared against an intercept-only (*-*) model to evaluate their respective explanatory power. We used Akaike’s information criterion with a correction for small sample sizes (AIC_*c*_) to compare fitted models, selecting formulations with the lowest AIC_*c*_ values as the best or most parsimonious model upon which further inferences were based [89]. Finally, for the selected model(s), we estimated the proportion of time bears spent resting versus traveling using the Viterbi algorithm, which provides the most likely sequence of unobserved behavioural states given the data and attendant fitted models [86, 88].

## Results

We obtained 33,699 locations from 58 individuals equipped with ear or fur tags between 2016 and 2022. Fur tags were affixed to 1 subadult and 15 adult male polar bears; 6 bears were equipped with pentagon tags, 6 were equipped with SeaTrkr tags, and the remaining 4 were equipped with tribrush tags. Ear tags were deployed on 5 subadult and 23 adult males between 2017 and 2022 on the Hudson Bay sea ice, and an additional 16 (subadult: *n* = *5;* adult: *n* = 11) were deployed on bears along the coast near Churchill, Manitoba between 2016 and 2021. Two adult males were recaptured during the study period and equipped with a second ear tag after their initial tag stopped transmitting, resulting in a total of 42 unique ear-tagged bears. All of the fur tags switched to inactivity mode, indicating that they became detached before the receivers stopped functioning, whereas only 3 of the 44 (7%) ear tags switched to inactivity mode. The remaining 41 stopped transmitting due to either malfunction or battery exhaustion.

**Fig. 3:**
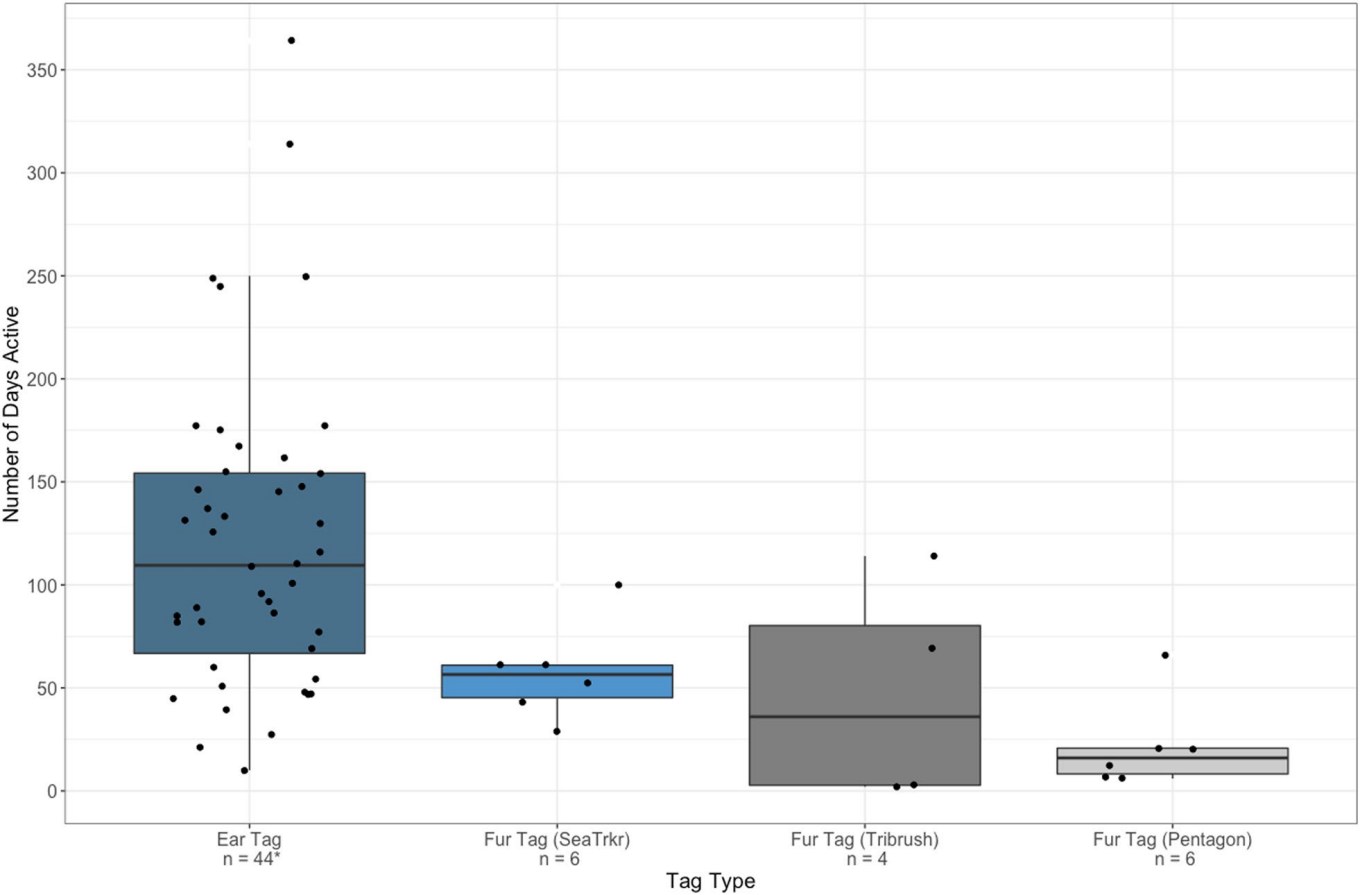
Number of days each type of ear and fur tag remained active while attached to a free-ranging subadult or adult male polar bear on the sea ice or coast of Hudson Bay between 2016 and 2022. Solid black lines represent the median duration of time each tag type remained active. Boxes show the interquartile range, spanning from the first quartile (lower edge) to the third quartile (upper edge). Whiskers extend 1.5 times the interquartile range above the third quartile and below the first quartile, whereas the data points beyond the whiskers are considered outliers. Data points are spread randomly along the x-axis to avoid overlap. *Two of the 42 bears equipped with ear tags between 2016 and 2022 were fitted with a subsequent ear tag after the first stopped functioning

Functional duration varied considerably both within and among the three different fur tag designs (Fig. 3).

SeaTrkr tags had the longest mean functional duration of the three fur tag designs, averaging 58 days (range: 29–100 days; SE: 9.8), whereas tribrush tags remained active for a mean of 47 days (range: 2–114 days; SE: 27.3). The two longest-lasting tribrush tags (69 and 114 days; mean: 91.5; SE: 22.5) were the only tags applied with supplementary adhesive. The remaining two tribrush tags that were applied without adhesive lasted an average of 2.5 days (range: 2–3 days; SE: 0.5). Pentagon tags had the shortest mean functional duration of the fur-tag designs, averaging 22 days (range: 6–66 days; SE: 9.2). Ear tags remained active for the longest mean duration of 121 days (range: 10–364 days; SE: 11.5). Five ear tags (11%) lasted > 200 days.

**Fig. 4:**
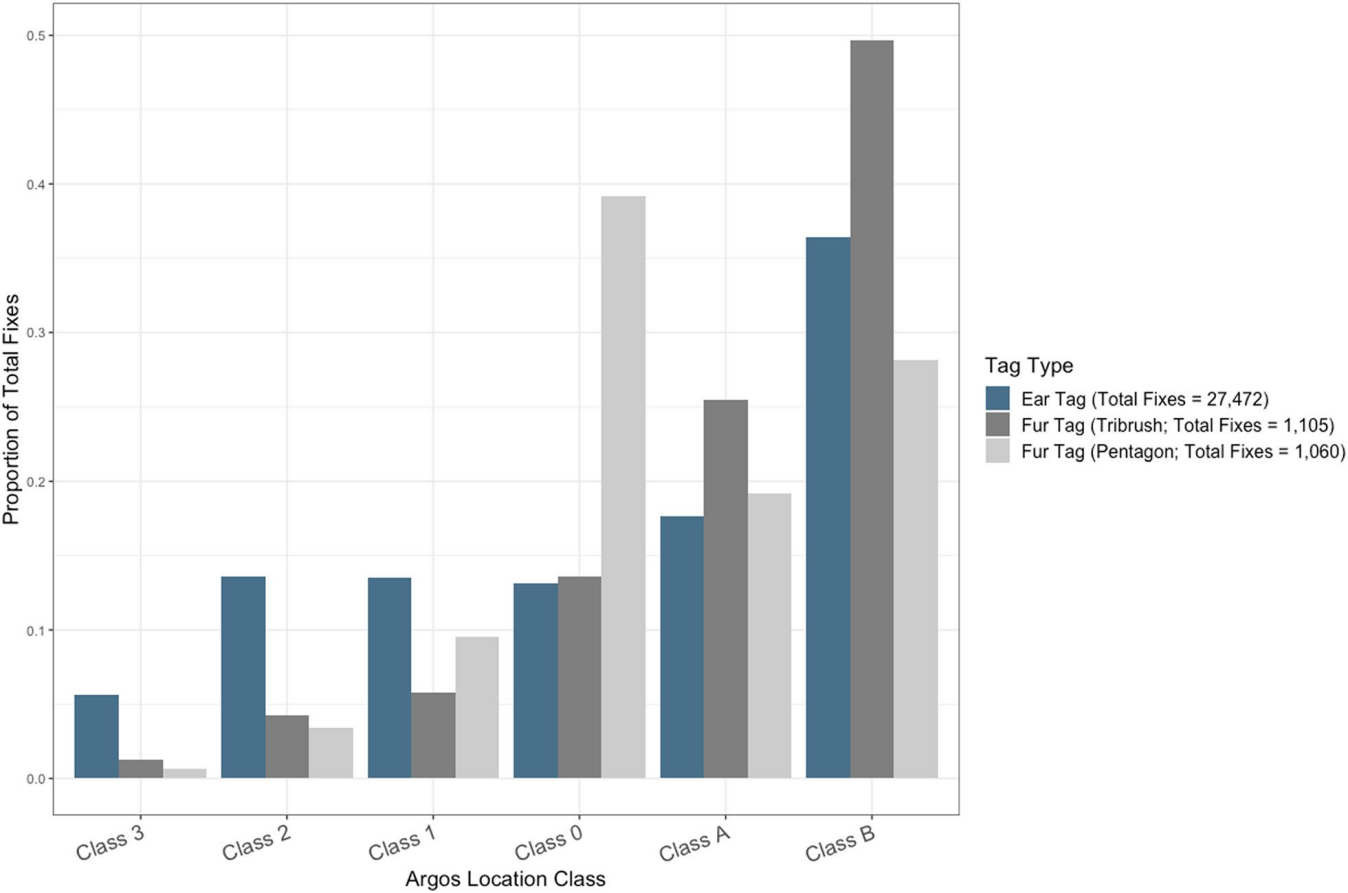
Proportion of fixes with each of the six location error classes for Argos-linked ear and fur tags deployed on subadult and adult male polar bears on the sea ice or coast of Hudson Bay between 2016 and 2022. Argos locations are assigned one of six error class estimates (3, 2, 1, 0, A, B; in order of decreasing accuracy) based on the number of messages transmitted to satellites. Locations derived from ≥ 4 messages have small horizontal error (< 250 m for Class 3, 500 m for Class 2, 1500 m for Class 1, and > 1500 m for Class 0), while those derived from < 4 messages cannot be estimated

Pentagon tags had a high proportion of fixes (39%) with Class 0 (> 1500 m) Argos location error, the largest of the Argos error classes. The remaining fixes had horizontal error estimates that were either < 1500 m (i.e., Class 1–3: 14%) or could not be estimated (i.e., Class A and B: 47%; see Fig. 4).

Tribrush tags, which used the same Argos receiver as the pentagon tags but had a protective radome covering, had the highest proportion of fixes for which horizontal error could not be estimated (Class A and B: 75%), followed by fixes with Class 0 (14%) and finally Class 1–3 (Class 1: 6%; Class 2: 4%; Class 3: 1%). Ear tags had the highest proportion of fixes with horizontal error estimates < 1500 m with fairly uniform proportions of Class 0–2 (Class 0: 13%; Class 1: 14%; Class 2: 14%) and A (18%) horizontal errors, and smaller and larger proportions of Class 3 (6%) and B errors (36%), respectively. Locations recorded using the six GPS/Iridium-equipped SeaTrkr tags had considerably smaller horizontal error values (mean = 11 m, range 4–102 m) than the Argos-linked tags.

Fur tags accounted for 6,227 locations and ear tags accounted for the remaining 27,472 locations of the combined 33,699 recorded between 2016 and 2022 (Fig. S1). Truncating data 3 day post-capture resulted in the removal of 420 and 909 locations from bears equipped with fur and ear tags, respectively. Filtering with the argosfilter R package removed 2,071 spurious locations, which accounted for *ca.* 7% of the combined data collected using Argos-linked ear and fur tags. Additional 18,300 on-ice locations were removed. After segmenting tracks with temporal gaps > 24 h and omitting those with < 100 locations, missing locations were interpolated, resulting in a total of 2,470 locations across 10 tracks from 9 individual bears, of which SeaTrkr tags accounted for 1,659 locations (6 tracks) and spring-deployed ear tags accounted for the remaining 811 locations (4 tracks). Argos-equipped fur and fall deployed-ear tags had insufficient data for inclusion in the HMMs.

**Fig. 5:**
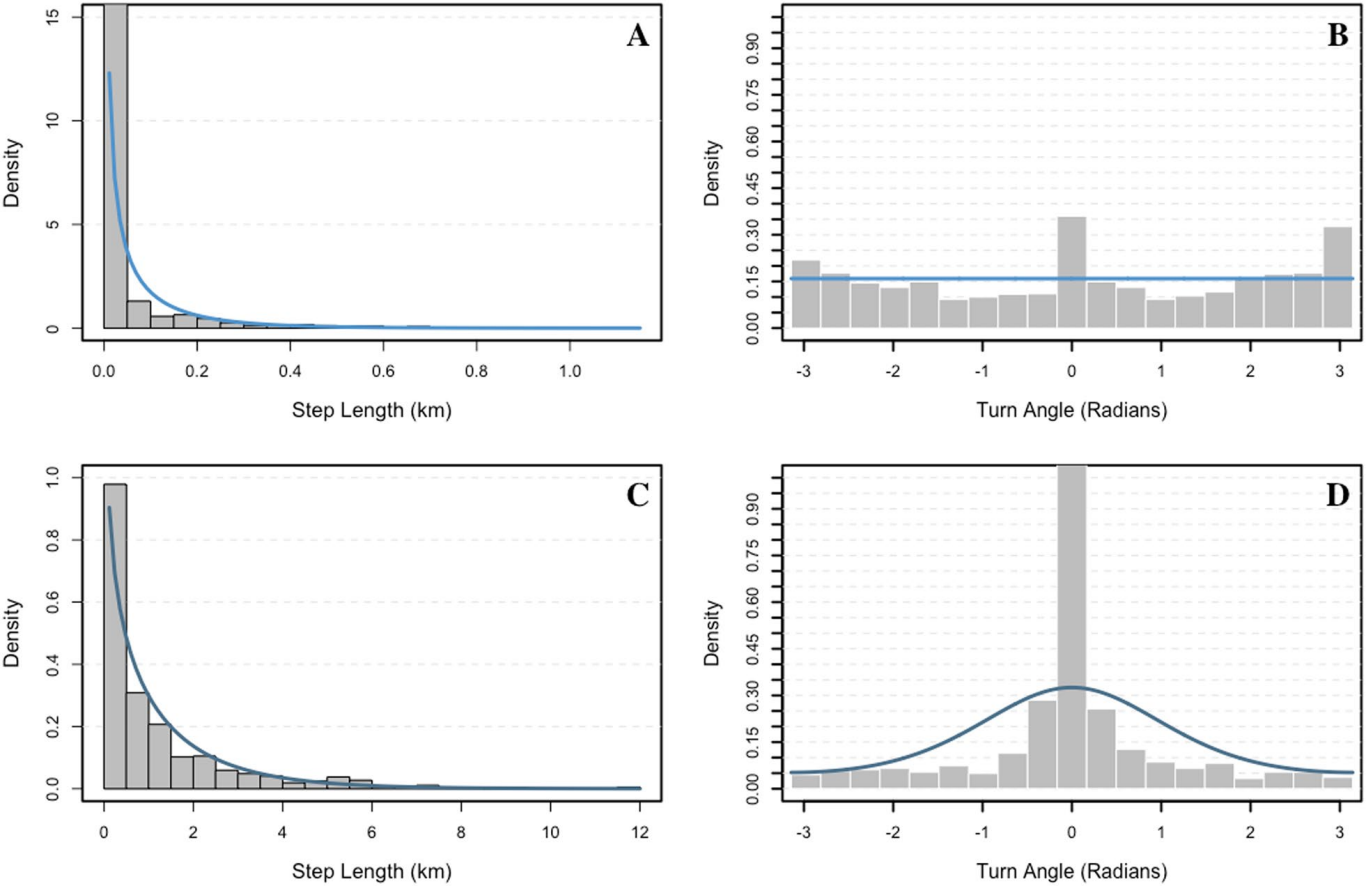
Results from two-state hidden Markov models developed for free-ranging subadult and adult male polar bears (9 individuals; 10 tracks) while on land equipped with Argos- and GPS/Iridium-linked ear and fur tags on the sea ice or along the Hudson Bay coast between 2016 and 2021. Step length (**A**) and turn angle (**B**) distributions for state 1 (resting), and step length (**C**) and turn angle (**D**) distributions for state 2 (travelling) overlaid atop step length and turn angle frequencies for combined data. Note the different scales used to display step length distributions

**Fig. 6:**
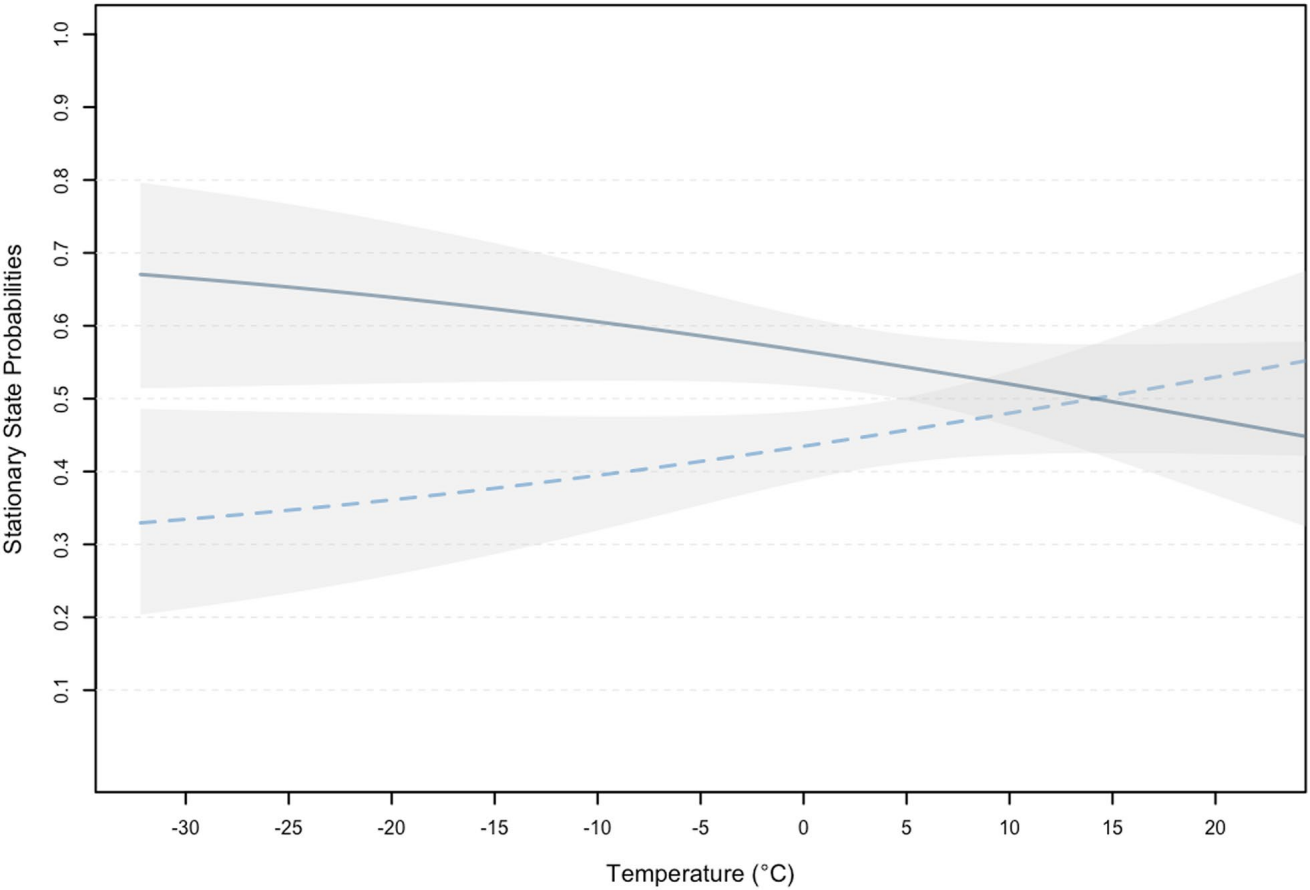
Stationary state probabilities for state 1 (resting; dashed light blue) and state 2 (traveling; solid dark blue) with 95% confidence intervals (grey bands) relative to ambient temperature from hidden Markov model developed for free-ranging subadult and adult male polar bears (9 individuals; 10 tracks) while on land equipped with Argos- and GPS/Iridium-linked and ear and fur tags on the sea ice or along the Hudson Bay coast between 2016 and 2022

Despite individual variation in movement characteristics and differences in tag retention times, two-state HMMs reliably differentiated among two behavioural states. The presumed resting state was characterized by short step lengths (mean: 0.01 km; 0.00 km/h; Fig. 5) and turn angles with greater concentration around π and – π, whereas the presumed traveling state was characterized by longer mean step lengths (mean: 0.74 km; 0.19 km/h) and turn angles concentrated closer to 0, suggesting greater directional persistence among successive locations. Based on model selection using AIC_*c*_, the two-state HMM that included linear effects of ambient temperature on state transition probabilities was deemed more parsimonious than the remaining models that included a polynomial term for ambient temperature and no temperature covariates (Table 1), suggesting that bears travelled more as temperatures cooled (Fig. 6).

**Table 1:** Model selection of two-state hidden Markov models fit to terrestrial movements of subadult and adult male polar bears (9 individuals; 10 tracks) equipped with Argos- and GPS/Iridium-linked ear and fur tags on the sea ice or along coast of Hudson Bay between 2016 and 2022

Model	K	AIC_c_	ΔAIC_c_	Log Likelihood
*temp*	13	2327.796	0	−1164.898
–	11	2338.155	10.359	−1170.078
*temp* + *temp*^*2*^	15	2826.065	498.269	−1414.032

Models are defined in the *Methods* section; *K* is the number of parameters in the model, and ΔAIC_c_ is the Akaike information criterion of each model relative to the best fitting or most parsimonious model with the lowest AIC_c_ score. The intercept-only model (-) included no effect of temperature on state transition probabilities

Finally, results from the Viterbi algorithm used to estimate the most likely sequence of unobserved behavioural states given the top-ranking model suggested bears spent 70% of their time resting and 30% of their time traveling while on land.

## Discussion

For decades, telemetry collars have remained the primary means of collecting long-term, high-resolution movement data from adult female polar bears [47]. However, the need to collect movement data from other age- and sex-classes of polar bears, together with a desire to provide alternatives to collars, particularly for certain short-term applications, led to the development of novel telemetry devices, including the fur tags we described and tested. Although fur tags had shorter mean functional durations than ear tags, they had similar horizontal error estimates and one of the designs provided sufficient data to quantify behavioural states of free-ranging subadult and adult male polar bears.

Although the specific causes of tag detachment are unknown, there are several aspects of polar bear behaviour that may have contributed to the short functional durations observed for fur tags, all of which became detached before battery exhaustion. While onshore, subadult and adult male polar bears generally remain close to the coast where they form aggregations and rest in shallow earthen pits [67, 90, 91]. Bears are frequently observed lying in a prone position, but routinely rest in lateral or supine positions as well. Between bouts of resting, bears occasionally swim in Hudson Bay or travel further inland, where coastal tundra transitions to areas dominated by taller vegetation, including willow and black spruce [39, 67, 92]. Thus, fur-mounted tags may be subjected to shear forces from ocean waves and abrasion against both sand and occasionally terrestrial vegetation. Male polar bears also engage in social play [90], including grappling and wrestling, which involve bears biting and wrapping their forelegs around the neck and/or shoulder region of their partner. Therefore, fur tags may also be susceptible to detachment during social play, because unlike ear tags, they were not permanently secured through an appendage but rather affixed to a more exposed part of the body.

Compared to ear tags and collars, fur tags were designed to remain affixed to polar bears for a relatively short period, as polar bear fur is replaced annually during a gradual moult between May and August [93–95]. Therefore, the maximum duration any fur-mounted tag can remain affixed to a free-ranging polar bear is approximately 1 year, after which it will be shed. Given the timing of this study (September to December), moulting likely did not contribute to premature detachment; however, ongoing hair growth may render the tags more prone to detachment over time. Thus, timing of application remains a consideration for future deployments, particularly during the spring and summer.

Differences in mean functional duration among the three fur tag designs are likely attributable to the method of attachment. For instance, SeaTrkr and pentagon tags were both attached using copper ferules crimped around multiple tufts of hair; however, SeaTrkr tags were secured to ten separate tufts of hair, while pentagon tags were only secured to five. Considering SeaTrkr tags are approximately double the size and nearly four times the weight of pentagon tags, it appears doubling the number of attachment points (i.e., 10 versus 5) contributed to the longer mean retention times for the larger SeaTrkr tags. In addition, SeaTrkr tags include a smooth outer casing, designed to reduce drag, whereas the Argos transmitters attached to pentagon tags remained exposed, secured to an accessory bolt on the tags using a nut and locking washer. Wiig et al. [56] noted several observations of polar bears with pieces of discarded fishing net caught on plastic identification ear tags, which are smaller than the Argos transmitters. The authors also speculate that instances of lost ear tag transmitters may be the result of similar entanglements. Thus, the more streamlined design of the SeaTrkr tags may have rendered them less likely than the irregularly shaped, multi-part pentagon tags to become caught in surrounding objects, or inadvertently removed by conspecifics during sparring.

Tribrush tags were attached by entangling hairs in three nylon-bristle pipe brushes that were twisted repeatedly inside perforated tubes spanning the length of the tags’ edges. Although care was taken during application to ensnare as much hair as possible, the relatively short coats of bears in late summer may have contributed to the tags’ short functional duration. Without adequate contact between the bristles and hair, tags may have loosened as the bears moved, leaving them susceptible to detachment. Application of supplementary adhesive appears to have enhanced retention time, as tribrush tags applied using two-part epoxy lasted 69 and 114 days, the longest duration of all the fur tags. Indeed, glue-on tags applied to polar bears in the Chukchi Sea provided location data for approximately 3 months [58]. The remaining two tribrush tags, which were applied without epoxy, lasted only 2 and 3 days. Although the exothermic reaction of the two-part epoxy caused concerns about potential damage to the hair and/or skin, a more targeted, lower volume application likely could ameliorate these issues. Unlike fur tags, which are designed to remain affixed for a short period, ear tags may remain attached indefinitely, because they are mounted through a hole in the ear and do not include a drop-off mechanism. The two bears that received a second ear tag after their first tag stopped transmitting were both recaptured with their initial, non-functional ear tag still attached. Thus, their size, means of attachment, and peripheral location likely make ear tags less prone to incidental detachment than fur tags, which are only secured to hair that may be shed or break on an exposed part of the bears’ torso. Fur tags are less likely to cause injury to the bear if they become entangled in debris or are pulled by another bear during social interactions, whereas ear tags, if entangled or pulled could cause injury.

Animal location data recorded using GPS receivers are usually accurate to < 20 m, whereas horizontal error estimates associated with data collected using Argos transmitters can only be specified to < 250 m [9, 75]. Accordingly, differences between GPS and Argos systems in terms of how location data are recorded is likely responsible for the higher resolution horizontal error estimates associated with the SeaTrkr GPS/Iridium tags compared to the Argos-linked tags.

Among the Argos-linked transmitters, tribrush tags had the largest proportion of fixes for which horizontal error could not be estimated (i.e., Class A and B). Transmitters were placed in the same position on each of the bears fitted with the pentagon and tribrush tags. However, in addition to slightly different means of attachment, tribrush tags were equipped with plastic radome covers, which were intended to help protect the transmitters from wind, precipitation, and abrasion. Pentagon tags did not include a protective covering, because the base was made of a flexible material that was less amenable to a cover. While radome covers are meant to have a minimal effect on the attenuation of electromagnetic signals, research using stationary GPS arrays has shown they may reduce the accuracy of GPS position estimates, particularly along the vertical axis [96]. The magnitude of signal attenuation also depends on the antenna design, along with the composition and thickness of the cover [96, 97]. Thus, the radome covers may have degraded the transmitters’ ability to reliably communicate with satellites, perhaps contributing to the higher proportion of inestimable horizontal error values associated with tribrush tags, a trend not observed in the nearly identical pentagon tags. Both pentagon tags and ear tags had similar proportions of fixes with Class 0–3 error estimates despite differences in attachment. Wiig et al. [56] reported similar proportions of fixes with high resolution error estimates for Argos-equipped SPOT-227 and -305A ear tags (mean: 64.68% and 53.10%, respectively) deployed on subadult and adult polar bears of both sexes. While male polar bears may spend extended periods during the ice-free season lying in shallow earthen pits, often excavated in coastal ridges [91, 98], it appears fur and ear tags remained comparably unobscured, resulting in similar proportions of successful transmissions to Argos satellites. The consistently low horizontal error estimates associated with GPS/Iridium-linked SeaTrkr tags further suggest that fur tags positioned on a bear’s back provide adequate communication with satellites and comparable error resolution to conventional GPS collars. The high positional accuracy of the SeaTrkr tags, along with their longer mean retention time, suggest that these tags may provide the best option among the three fur-mounted tag designs for tracking polar bear movements over short time periods.

Our results demonstrate that subadult and adult male polar bears limit their movements while ashore, corroborating previous observational studies that showed bears spent approximately 70–90% of their time resting during the ice-free period in Hudson Bay [90, 99, 100]. Polar bears spend similar proportions of time inactive while on the spring sea ice where they often adopt a sit-and-wait hunting style [101–103]. During the spring, bears must consume one adult or subadult seal every 5 days to avoid energy deficit [103]. While ashore, polar bears do not have access to energy-rich marine mammal prey. Instead, they rely on stored fat reserves and terrestrial food sources that provide limited nutritional contributions, resulting in losses of approximately 1.0 kg of body mass per day [91, 104–106]. Given their inability to offset energy loss through food acquisition, we expected the observed proportion of time bears remained inactive to be higher. However, as hypothesized, bears varied their activity in accordance with ambient temperature. Indeed, the top-ranking two-state HMM, which included a linear effect of ambient temperature on stationary state probabilities, suggested that bears travelled less during warmer weather, increasing their time spent traveling as temperatures cooled. Colder temperatures coincide with sea ice formation during the late fall and early winter in Hudson Bay when all polar bears, with the exception of pregnant females, increase movement rates as they begin a seasonal migration towards newly forming sea ice [27, 107, 108]. If, as predicted, the duration of the ice-free season in Hudson Bay continues to increase, bears may need to further reduce activity levels to offset prolonged fasting, or risk further declines in body condition and higher mortality due to starvation [106, 109–111].

We only considered two behavioural states due to the limited temporal resolution and number of locations recorded using our tags. The GPS/Iridium-linked SeaTrkr tags consistently provided reliable location data, whereas locations from the Argos-equipped ear and fur tags were more irregular, resulting in a high proportion of data loss due to filtering and regularization. Accordingly, locations from the SeaTrkr tags comprised the majority of data used to fit the HMMs. Given these data limitations, along with research suggesting bears spend only *ca.* 3% of their overall time budget foraging while on land, it is unlikely HMMs could reliably distinguish between three or more distinct states [48, 51, 99]. Others have demonstrated polar bears engage in additional behaviours, particularly during winter and spring while on the sea ice [51, 99, 112, 113]. For instance, Togunov et al. (2022), using a similar HMM approach, showed adult female bears alternated between 3 distinct movement-related behavioural states (drifting, area restricted search, and olfactory search) while on the sea ice between January and June. Similarly, Pagano et al. [51] showed video-linked accelerometer data collected at 2-s intervals could be used to distinguish between 3 behaviours (i.e., resting, walking, and swimming), and were capable of identifying up to 5 behaviours while bears were on land (i.e., resting, walking, eating, grooming, and head shaking). Future behavioural studies using fur tags, particularly GPS/Iridium-linked tags, may consider increasing fix-rates to identify more behaviours and attendant habitat associations.

While collars remain the best option for collecting long-term, high-resolution movement data from adult female polar bears [44], fur-mounted tags offer promise for short-term applications, particularly for bears of other sex- and age-classes, and situations where collars may be inappropriate (e.g., stakeholder opposition and/or monitoring problem bears). For instance, fur tags may be used to study the movements and behaviours of polar bears during important periods, such as the spring hyperphagia and mating seasons, transition on and off the sea ice, and during the ice-free season. Short-term monitoring of subadult and adult males may further clarify sex- and age-class-related differences in movement characteristics, including home range size and habitat selection. Furthermore, fur tags may be well-suited for use in mitigation of human-bear conflicts. Bears captured near human settlements could be equipped with the temporary tags to monitor their proximity to people and infrastructure, allowing conservation staff to intercept the bears and prevent recidivist encounters. GPS collars are poorly suited for this singular task because most bears involved in conflicts are subadult and adult males [72, 73, 114, 115]. Fur tags offer promise as a safe, short-term means of monitoring the movements of free-ranging polar bears for purposes of both applied scientific research and mitigating human-bear conflicts. SeaTrkr tags in particular provide the benefits of secure but temporary attachment, relatively long functional duration, high spatial error resolution, and consistent location data, all of which are important factors to consider when selecting wildlife tracking devices.

Further refinement and testing of fur tag designs may improve their reliability. Our results demonstrate that increasing the number of attachment points, along with use of suitable supplementary adhesive, ought to increase mean retention times. Further tests may also be used to evaluate their suitability for use on other age classes. Tracking bears other than adult females is important for understanding critical aspects of the species’ habitat use and behaviour, particularly with ongoing climate change. Current estimates suggest that climate-mediated changes to Arctic environments are likely to cause shifts in polar bear distribution and habitat selection, and result in higher rates of human-bear conflicts [23, 73, 110, 116]. Therefore, along with other remote tracking technologies, including ear tags, fur-mounted tags offer a means of collecting data that will enable managers and other stakeholders to make informed decisions important for the ongoing management and conservation of polar bears.

### Supplementary Information

Supplementary material 1.

## Data Availability

The data that support the findings of this study are available from the corresponding author upon reasonable request.
