# Supplementary material for: Telemetry without collars: performance of fur- and ear-mounted satellite tags for evaluating the movement and behaviour of polar bears
Source: Anim Biotelemetry. 2024 Jul 15;12(1):18. doi: 10.1186/s40317-024-00373-2 (PMC11249465; doi:10.1186/s40317-024-00373-2)

**Figure S1**. Sample sizes for ear and fur tags applied to free-ranging subadult or adult male polar bear on the sea ice or coast of Hudson Bay between 2016-2022 corresponding to data truncation and filtering procedures applied before fitting hidden Markov models. The original, full suite of non-truncated and non-filtered data were used to determine the tags’ functional duration and horizontal error values.
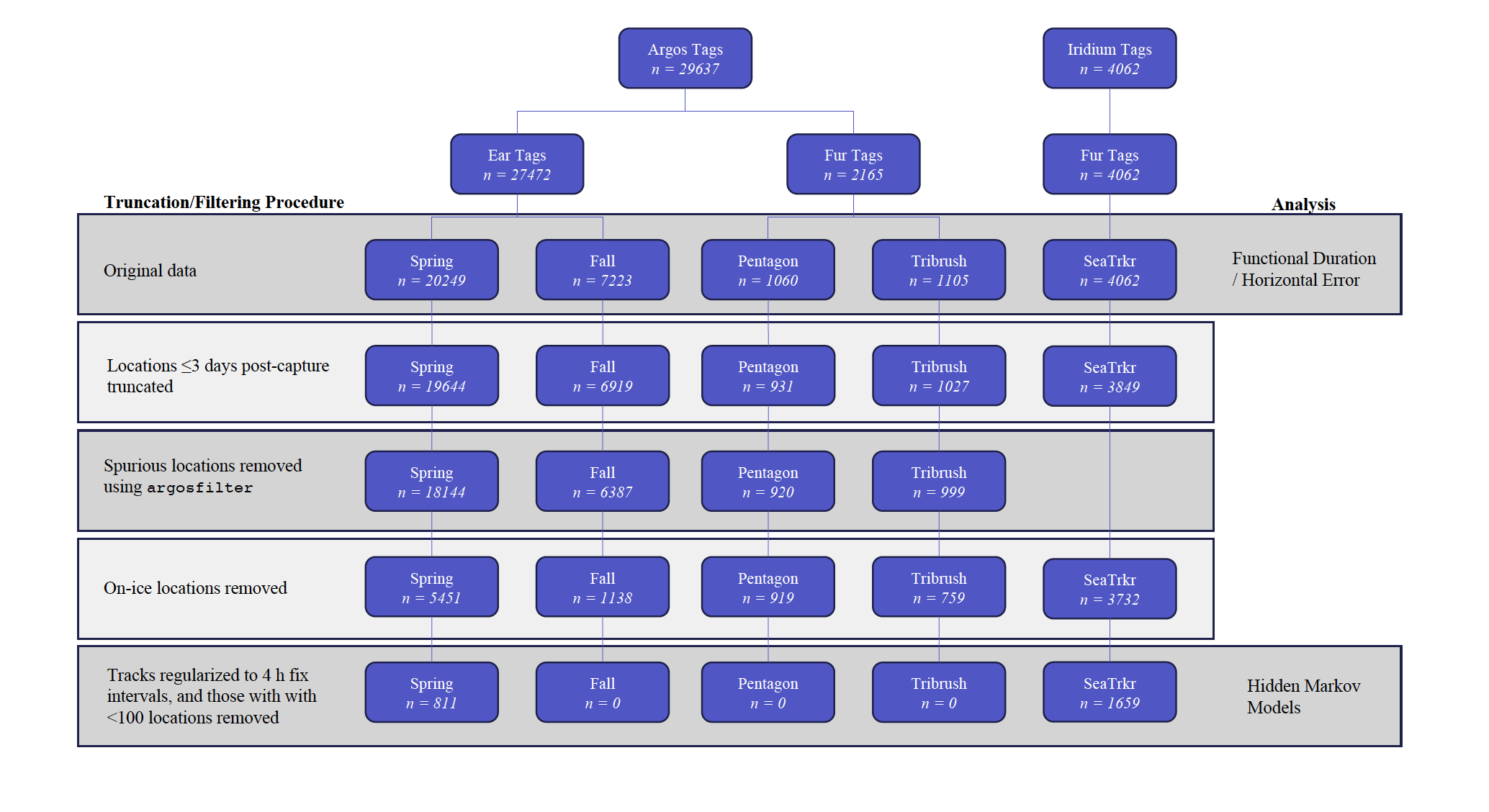

Supplement: Supplementary file 1 — Supplementary material 1. [file 40317_2024_373_MOESM1_ESM.docx]
